# Lack of recognition at the societal level heightens turnover considerations among Nordic eldercare workers: a quantitative analysis of survey data

**DOI:** 10.1186/s12913-021-06734-4

**Published:** 2021-07-27

**Authors:** Jon Ivar Elstad, Mia Vabø

**Affiliations:** NOVA, Centre of Welfare and Labour Research, Oslo Metropolitan University, P.O.Box 4, St. Olavs Plass, 0130 Oslo, Norway

**Keywords:** Elderly care, Staff turnover, Social recognition, Questionnaire, Linear probability models

## Abstract

**Background:**

Recruiting and retaining staff are standing challenges in eldercare. Low pay, difficult working conditions, and social relations at the workplace impact on turnover intentions. Few studies have used quantitative data for estimating the role of recognition by the wider society for staff instability. This study examines how perceived lack of recognition at the societal level affects Nordic eldercare workers’ considerations of leaving their jobs.

**Methods:**

The 2015 Nordcare survey among frontline eldercare workers in Denmark, Finland, Norway, and Sweden (*N* = 3,677) is analysed. Issues such as working conditions, financial strain, work-life balance, and appreciation by care recipients and colleagues, were covered. Recognition at the societal level was measured by perceptions of being valued by top municipal leaders, mass media, and the general public. Analyses are made with cross-tabulations and multivariate linear probability regression models.

**Results:**

In the total sample, 41.1 % had “seriously considered to quit during the last 12 months”. About one third felt “not at all valued” by top municipal leaders, while one fourth felt “not at all valued” by mass media. In bivariate analyses, perceptions of recognition were strongly associated with considerations to quit. These associations were reduced, but remained sizeable and highly significant in multivariate analyses adjusted for age, gender, health, working conditions, financial stress, workplace relations, and other known turnover predictors.

**Conclusions:**

Lack of recognition by societal agents such as top municipal leaders, mass media, and the general public, is widely felt by Nordic eldercare workers. Feeling poorly valued by such sources is associated with frequent considerations to leave one’s employment. Perceived lack of recognition by the wider society has a significant and independent impact on staff instability in the eldercare sector. Societies’ recognition order is embedded in social structures which are resistant to change, but policies which succeed in raising the societal recognition of eldercare work may contribute to reduced retention difficulties in eldercare.

**Supplementary Information:**

The online version contains supplementary material available at 10.1186/s12913-021-06734-4.

## Background

With population ageing, recruitment and retention of eldercare staff are urgent policy challenges. A recent report [1] found that in three quarters of OECD countries, the growth in number of older adults in need of long-term care is outpacing the eldercare workforce (p.14). Both attracting and keeping staff face difficulties. In the 24 analysed OECD countries, average tenure in the long-term care sector was two years shorter than the average for other sectors (p.99). Turnover and understaffing threaten care quality and institutional efficiency [2–4]. Recipients will benefit from stable relationships to the same care workers; staff instability obstructs building good work routines; and frequent replacement of workers requires considerable administrative resources.

Recruitment and retention problems in eldercare are associated with low pay, poor career prospects, non-standard working hours, physically hard and mentally exhausting work, time pressure, and insufficient training [1]. Typically, wages are not only markedly below the country average, but also below pay levels in similar hospital work (p.10). Lack of promotional opportunities [5], a strenuous workload [6, 7], and mental strain when caring for patients with dementia [8], can elevate turnover risk. Working at evenings, nights, and weekends complicates work-life balance ([9, 10]; Drange I, Vabø M: A cross-sectional study of sustainable employment in Nordic eldercare, forthcoming). Part-time and temporary employment generate financial stress [11], and austerity and marketisation reforms can engender job insecurity [12, 13].

Eldercare work can nevertheless be experienced as satisfying and intrinsically rewarding [14, 15]. It corresponds to shared moral obligations that society should take care of frail elderly. Praise from recipients and their relatives make eldercare workers feel appreciated. Job satisfaction, job commitment, and staff stability are boosted when eldercare workers have good relations with clients and co-workers and experience support from superiors [4, 7, 16].

Such observations point to the relevance of *recognition* for retention problems in eldercare. Recognition, in short, means being seen, heard, valued, and taken into account [17, 18]. Recognition communicates acknowledgement of a person and approval of her contribution. It signifies esteem and respect – in contrast to *misrecognition*, i.e., being unnoticed, neglected, and disregarded. Recognition theorists have slightly different views on typical effects of misrecognition. For Axel Honneth, lack of recognition is a psychological injury which impedes self-realisation and deprives a person from satisfying the vital human need of being noted, accepted, and loved [19]. For Nancy Fraser, lack of recognition is an institutionalised status subordination which excludes individuals and groups from “participatory parity” and denies their inclusion as “full members of society” [20](p.31). Both versions are relevant for the retention challenge. They imply that lack of recognition devalues eldercare work and belittles eldercare workers’ contribution to a common social project, resulting in reduced work motivation and heightened turnover [21, 22].

Recognition is a common theme in management theories [22, 23] and management manuals [24, 25]. They advise that staff stability will benefit from a deliberate utilisation of recognition tools such as individualised support, public praise of employees’ performance, and attention to their personal well-being. These themes are also prominent in research on recruitment and retention challenges in health care. Studies recommend that care managers should consciously and publicly display their appreciation of the staff [26], for instance by “staff recognition initiatives” such as long-term service awards, annual BBQs, and holiday parties [27]. Empirical evidence underwrites such recommendations, as studies have shown that support and approval from work colleagues as well as praise and recognition from close superiors are associated with less turnover and improved staff stability [28–30].

Typically, such studies focus on recognition at the workplace. The feeling of being appreciated and esteemed in day-to-day interaction with care recipients, work colleagues, and close superiors, has been highlighted [4]. However, also recognition outside of the workplace may be highly relevant for the retention challenge in eldercare. Studies have shown that perceptions of being valued in the local community are associated with job satisfaction, work motivation, and less turnover intentions [21, 31].

At the *societal* level, in the wider society beyond the local community, also other sources and agents may contribute to recognition – or misrecognition – of eldercare work. Such work is typically characterised as low status [17, 18] and assigned little prestige [32]. This unfavourable placement in the occupational hierarchy does not only allude to low wages and problematic work conditions such as exposure to violence [33], exhausting emotional demands [8], and time pressure [15]. The low status of eldercare work is also linked to disregard, little respect, and a low overall standing in society. Unfortunate consequences are underlined in a British study based on qualitative interviews with employees in care organisations [34]. Respondents mentioned frequently the low status of eldercare work as reasons for staff instability, and expressed a need for making care work more treasured by “communities and public systems” (p.14). The recent OECD report [1] argues similarly that retention problems in eldercare are partly due to prevailing images of eldercare work. Advertisements and public campaigns aiming at improving the image of this sector are proposed as promising measures which may elevate the status of eldercare work and improve the self-concept of eldercare workers (p.50).

### Purpose and hypothesis

The purpose of the present study is to explore further the role of recognition at the *societal* level for the retention challenge in eldercare. Our hypothesis is that lack of recognition and poor valuation of eldercare work by the wider society has a sizeable impact on staff instability and turnover considerations among eldercare workers. Various reports, policy statements, and studies based on qualitative interviews with care personnel point in this direction [1, 18, 34]. However, statistical and quantitative investigations of the topic appear to be very scarce. An exception is an Australian study based on a large survey of eldercare workers [21]. It showed that perceptions of lack of recognition in the local community contributed significantly to contemplations about leaving their job, after adjusting for wage levels, work conditions, and social relations in one’s care organisation. We have not found any similar study that estimates the importance, in quantitative terms, of recognition by the wider society, beyond the local community, on staff instability and turnover intentions in the eldercare sector.

**Fig. 1:**
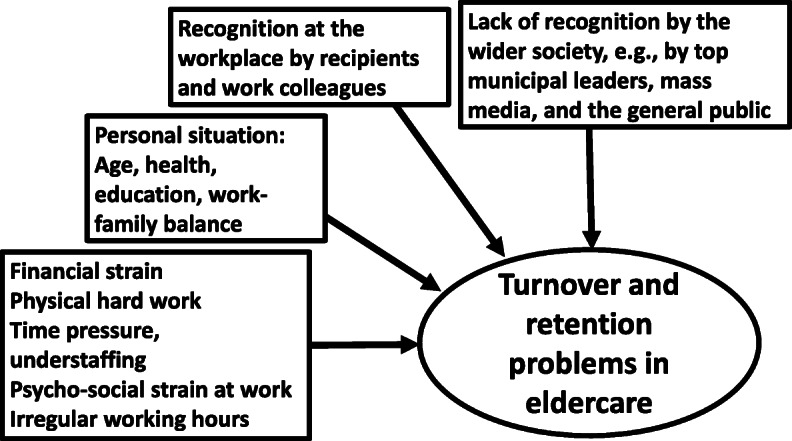
The hypothesis

The present study contributes to fill this knowledge gap by analysing survey data obtained from a large sample of frontline eldercare workers in four Nordic welfare states. Workers’ perceptions of recognition at the societal level are measured by three indicators which represent different agents in the wider society: top leaders in the municipality, mass media, and the general public. We examine the associations between these indicators and considerations to quit employment in eldercare. We hypothesise that lack of recognition from the wider society plays a significant and *independent* role. Thus, lack of recognition at the societal level is not just a side effect of low pay, poor working conditions, and valuation at the workplace. Rather, perceived lack of recognition from distant sources at the societal level influences staff stability and turnover considerations in a unique and autonomous way, over and above the effects of other well-known determinants. Figure 1 illustrates the hypothesis.

## Methods

### Data and main variables

In the Nordcare 2015 survey, practically identical questionnaires (translated into each country’s language) were mailed to unionised care workers in the four Nordic countries Denmark, Finland, Norway, and Sweden. Respondents were randomly chosen from the membership registers of the unions which organise practical nurses, care aides, home helpers, and similar frontline care occupations. Those with current or recent employment in the eldercare sector were invited to participate. Respondents were primarily working in nursing homes or in home care services for the elderly. In the Nordic welfare states, eldercare institutions are mostly owned, managed, and funded by the municipalities, but some are owned by non-profit “ideal” organisations or by commercial enterprises [35], although largely financed by municipal subsidies.

In total, 3,677 answers were registered (1016 from Denmark, 972 from Finland, 920 from Norway, and 769 from Sweden). The great majority were female (about 96 % of the total sample), and 78 % were employed by municipalities. Overall response rate has been estimated to 55 % [36]. Missing answers on specific questions were few (seldom above 2 %).

The analysed outcome is respondents’ answer – yes or no – to the question: “Have you during the last 12 months seriously considered to quit your work?” A key word in the question is “seriously”. It suggests that answers will usually reflect sincere deliberations about staying on or leaving their job. Turnover intention does not necessarily lead to actual turnover, but since it predicts subsequent quitting to some degree [37, 38] answering “yes” to the question will indicate potential staff instability in the eldercare organisation.

Perceived recognition at the societal level was measured by three questions: “Do you think that your work is valued (1) by top municipal officials and politicians, (2) by the mass media, and (3) by the general public?” Feeling valued is an appropriate indicator of perceptions of recognition. Two questions with the same wording measured recognition at the workplace, referring to care recipients and to work colleagues. Answers were classified in three levels: “Yes, very much or quite much valued”, “No, not much valued”, and the curt response “*Not at all* valued”. In addition, “Don’t know”-answers are reported since quite a few chose this response alternative when asked about recognition at the societal level.

### Analyses

The key interest in this study is to examine the occurrence of perceived lack of recognition by the wider society, and to investigate the associations between recognition at the societal level and eldercare workers’ contemplations about quitting their job. These associations are first analysed by bivariate cross-tabulations.

In order to explore the *unique* effects of recognition at the societal level, an extensive set of control variables are included in multivariate analyses. Workplace recognition and recognition at the societal level might overlap. In order to estimate the effects of societal recognition *net of* workplace recognition, two variables representing recognition by care recipients and by work colleagues are included. Background control variables are country, gender, age (three levels: up to age 39 years, 40—54 years old, age 55 and above), and immigrant status (i.e., born abroad or born in the respective country). Two variables indicate respondents’ education: years of schooling in general education (three levels), and education in care work (three levels). Duration of work experience is measured by a four-level variable. No separate questions about wages were asked (pay levels vary considerable between the Nordic countries), but answers to the question “Are you worried that your earnings are insufficient for covering your living expenses?” indicate whether the respondents experienced financial strain due to low income. Assessment of work-life balance is indicated by answers to the question “How well are your work schedule/working hours suited to your family life and free-time activities?” Problematic work conditions are measured by an additive index based on questions about working paid overtime, working unpaid overtime, skipping lunch because of work stress, understaffing because of sickness or vacancies, unable to perform planned care tasks due to too much work, physically heavy work because of lifting or carrying, working in physically strenuous positions, and working alone on tasks that normally should require two workers. The index counts how many of these eight types of problematic work conditions were experienced daily or weekly. A similar additive index counts harassment by recipients or their relatives: daily or weekly experiences of yelling, violence/threats, sexual pestering, and racist or xenophobic remarks. Health problems are an important reason for considerations to quit, measured by a dichotomous variable – very good or good, versus fair or bad self-rated health. Appendix Table 1 describes the control variables in the four country samples.

The effects of perceived recognition at the societal level, adjusted for controls, are explored by linear probability regression models. Linear probability models were chosen since results are easier to interpret than the coefficients in the alternative logistic regression models, and the two techniques will usually give comparable estimates [39–41]. Testing showed that the results from linear probability models and logistic regression models corresponded closely (available from the authors). In order to avoid overburdening of the tables, 95 % confidence intervals are only reported for the linear probability models.

## Results

The upper part of Table 1 shows the distributions of feeling valued at the societal level. A substantial proportion felt “not at all valued” by top municipal officials and politicians: about one third of the samples in Denmark, Finland, and Sweden chose this response alternative (this question was not asked in the Norwegian sample). Quite few – varying from 7 to 11 % in the country samples – felt “very much/quite much valued”.

**Table 1:** Perceived recognition (%) at the societal level and at the workplace

Sample	Denmark	Finland	Norway	Sweden
*Recognition at the societal level*				
* Valued by top municipal officials/politicians?*				
- Yes, very much/quite much valued	11.4	7.0	*	9.3
- Not much valued	28.3	37.9	*	19.9
- Not at all valued	33.5	33.7	*	32.8
- Don’t know	26.7	21.4	*	38.1
*N* = 100.0 %	996	962	*	754
* Valued by mass media?*				
- Yes, very much/quite much valued	8.6	11.9	15.2	8.8
- Not much valued	33.9	41.5	34.6	22.4
- Not at all valued	35.6	23.1	18.9	32.3
- Don’t know	21.9	23.5	31.3	36.5
*N* = 100.0 %	990	958	882	750
* Valued by the general public?*				
- Yes, very much/quite much valued	20.3	36.9	45.0	27.9
- Not much valued	39.8	43.1	25.6	23.2
- Not at all valued	21.2	6.5	6.5	17.4
- Don’t know	18.6	13.5	23.0	31.5
*N* = 100.0 %	989	963	892	753
*Recognition at the workplace*				
* Valued by recipients?*				
- Yes, very much/quite much	91.5	87.3	95.3	94.6
- Not much	6.8	10.4	3.3	3.6
- Not at all valued	0.2	0.4	0.1	0.3
- Don’t know	1.5	1.9	1.2	1.6
*N* = 100.0 %	999	962	897	757
* Valued by work colleagues?*				
- Yes, very much/quite much	85.6	87.6	89.4	88.1
- Not much	10.9	8.3	6.9	7.3
- Not at all valued	0.8	0.8	0.3	0.9
- Don’t know	2.7	3.3	3.3	3.7
*N* = 100.0 %	999	964	897	756

*This question was not asked in the Norwegian survey

Also as to recognition by mass media, only a minority felt “very much/quite much valued” (varying from 9 to 15 %). In the Danish and Swedish samples, feeling “not at all valued” by the mass media were reported by 36 and 32 %, respectively, but this negative response was less prevalent in the Finnish (23 %) and Norwegian samples (19 %). Somewhat more positive responses emerged as to being valued by the general public, with considerable country variations: more positive answers in Finland and especially in Norway, than in Denmark and Sweden. In all country samples, “Don’t know”-answers about recognition at the societal level occurred frequently.

The answers as regards recognition at the workplace (the lower part of Table 1) were very different from the societal level answers. A very high percentage, varying from 87 to 95 % in the four countries, reported being “very much or quite much valued” by care recipients, while less than 0.5 % felt “not at all valued”. Similarly, 85–89 % felt “very much or quite much valued” by work colleagues, while less than 1 % used the “not at all valued” response. “Don’t know”-answers as to recognition at the workplace were very few.

The average percentage reporting “seriously considered to quit” was 42 % in Denmark, 38 % in Finland, 34 % in Norway, and 52 % in Sweden (see Appendix Table 1). Table 2 shows that turnover intentions increased substantially the more the respondents perceived lack of recognition from the wider society, in all four countries. In the Danish sample, for example, when feeling “very much/quite much valued” by top municipal officials and politicians, 23 % reported having seriously considered quitting, but this percentage rose to 59 % among those who felt “not at all valued”, i.e., a 36 % point difference. Marked differences in quitting considerations occurred also between ”very much/quite much” and “not at all” valued by mass media: 17 % point difference in Denmark, 20 in Finland, 18 in Norway, and a 27 % point difference Sweden. As to recognition by the general public, the percentage point difference between the “very much/quite much” and “not at all” categories was 20, 37, 14, and 25 in the Danish, Finnish, Norwegian, and Swedish samples, respectively. Those who answered “don’t know” about recognition from these sources tended to report turnover intentions fairly close to the average in the country sample.

**Table 2:** Considerations to quit, by level of perceived recognition at the societal level

Source of perceived recognition	Very/quite much valued	Not much valued	Not at all valued	Don’t know if valued or not
*Top municipal officials/politicians*^a^	Percentages reporting “seriously considered to quit”			
Denmark	23.4	37.5	58.8	33.7
Finland	15.4	33.8	52.9	30.5
Sweden	26.9	51.4	65.0	49.1
*Mass media*				
Denmark	35.7	38.1	52.9	34.0
Finland	31.5	37.5	51.6	29.5
Norway	24.4	37.6	41.9	29.0
Sweden	39.4	44.4	66.0	49.4
*General public*				
Denmark	35.9	42.7	55.6	32.0
Finland	29.9	40.8	67.2	38.3
Norway	28.8	41.7	42.9	31.5
Sweden	42.7	54.1	67.5	52.6

^a^The question was not asked in the Norwegian survey

Accordingly, Table 2 displays strong *bivariate* associations between the perceptions of being valued at the societal level and considerations to leave eldercare work. However, our hypothesis is that lack of recognition at the societal level adds significantly to staff instability *over and above* the effects of wage levels, work conditions, recognition at the workplace, and other well-known determinants of turnover intentions. Multivariate linear probability models, adjusting for such determinants, can shed light on this hypothesis. In these models, the four country samples are pooled, which is appropriate since the organisation of eldercare has many common features in the four countries [35], and the overall pattern of associations between recognition and quitting considerations was similar in all four samples.

Table 3 reports the estimated probability of “seriously considered to quit” among those who felt “not much” and “not at all” valued, relative to the reference category “very much/quite much valued”, separately for the three societal recognition indicators. Model 1 includes only the societal recognition variable, while Model 2 adds the control variables: perceptions of being valued by recipients and by work colleagues, country, and the other control variables listed above and described in Appendix Table 1. Table 3 reports only the results with relevance for the hypothesis; Appendix Table 2 shows the control variable coefficients when analysing recognition by mass media (the control variable coefficients were practically identical when analysing recognition by top municipal leaders and the general public).

**Table 3:** Linear probability models; outcome “serious considerations to quit” (no = 0, yes = 1)

	Model 1 – unadjusted			Model 2 – fully adjusted^a^		
	B	95 %CI	*p*-val	B	95 %CI	*p*-val
*Recognition by top municipal officials/politicians*						
Very/quite much valued (ref.)						
Not much valued	**0.156**	0.089/0.223	< 0.001	**0.088**	0.025/0.151	0.006
Not at all valued	**0.354**	0.289/0.420	< 0.001	**0.194**	0.131/0.257	< 0.001
Don’t know	**0.160**	0.093/0.227	< 0.001	**0.106**	0.043/0.169	0.001
Constant		0.230			0.318	
Adjusted R square		0.052			0.201	
N		2,645			2,645	
*Recognition by mass media*						
Very/quite much valued (ref.)						
Not much valued	**0.077**	0.024/0.129	0.004	0.019	-0.029/0.068	0.437
Not at all valued	**0.230**	0.176/0.285	< 0.001	**0.083**	0.032/0.134	0.001
Don’t know	0.051	-0.003/0.105	0.064	0.037	-0.013/0.087	0.146
Constant		0.310			0.355	
Adjusted R square		0.027			0.186	
N		3,527			3,527	
*Recognition by general public*						
Very/quite much valued (ref.)						
Not much valued	**0.107**	0.068/0.146	< 0.001	0.032	-0.005/0.062	0.092
Not at all valued	**0.261**	0.208/0.314	< 0.001	**0.091**	0.037/0.142	0.001
Don’t know	**0.067**	0.022/0.112	0.003	0.052	0.010/0.094	0.015
Constant		0.329			0.357	
Adjusted R square		0.026			0.186	
N		3,527			3,527	

*B = *unstandardised regression coefficient, *95 %CI = *95 % confidence interval, **bold** coefficients = *p*-value < 0.01

^a^Adjusted for recognition at one’s workplace, country, gender, age, immigrant status, general education, care education, work experience, financial strain, work-life balance, distressing working conditions (index constructed with 8 items), harassment at work (4 items), and self-rated health (see Appendix Table 2 for control variable coefficients)

Since no adjustment is made in Model 1, the coefficients for “not much” and “not at all” valued correspond to the results reported in Table 2. In the fully adjusted Model 2, these coefficients were markedly reduced, implying that the effects of perceived recognition at the societal level and the control variables overlap and correlate. Nonetheless, also the fully adjusted Model 2 indicates very clear associations between perceptions of recognition at the societal level and considerations to quit. Thus, as to recognition by top municipal officials and politicians, the initial Model 1 coefficient for “not at all” versus “very much/quite much” was 0.354, i.e., a 35.4 % point difference in probability of seriously considering quitting. In Model 2, this coefficient was reduced to 0.194, indicating an estimated difference of 19.4 % points in probability of quitting considerations between the two valuation categories – a substantial difference which is highly significant in statistical terms. The corresponding coefficients for recognition by mass media (0.083) and by the general public (0.091) are smaller, but highly statistically significant and relevant for the retention problem.

In sum, the results displayed in Table 3 are in line with the hypothesis that perceptions of recognition by the wider society have a unique and independent effect on these eldercare workers’ considerations to leave their jobs, over and above the combined effects of a broad range of other well-known determinants of staff instability.

## Summary and discussion

### Main findings

The retention challenge in Nordic eldercare is illustrated by the finding that about 40 % of the analysed eldercare workers, most of them with long previous careers in eldercare, answered that they had “seriously considered to quit” during the last 12 months. This study provides an analysis of how such considerations of leaving one’s eldercare job were related to perceptions of recognition.

The great majority – around 90 % of the analysed eldercare workers – felt “very much or quite much” valued at their workplace, i.e., by care recipients and by work colleagues. Perceptions of recognition at the societal level were markedly worse. Around one third of the total sample answered “not at all” valued by top municipal officials and politicians (Table 1). Negative perceptions of being valued by mass media were also common, but they were somewhat less prevalent as regards the general public. As to recognition at the workplace, “don’t know”-answers were very few, but when asked about recognition at the societal level, many chose the “don’t know”-alternative. A probable explanation is the frequency of experiences: eldercare workers would more seldom have direct encounters with agents at the societal level, while daily interactions at the workplace made it easier to form an opinion.

The particularly negative perception of recognition by top municipal officials and politicians (Table 1) is noteworthy. A likely reason is that experiences of problematic and deteriorating working conditions [4, 36] are associated with budget restraints and organisational changes implemented from above. Eldercare workers may assume that those in charge of the municipal eldercare sector have an obligation to provide decent working conditions and sufficient resources. Feeling that this obligation is not fulfilled can be experienced as a “contract violation” which nurtures not only frustration, but even contempt and cynical views on the top management and their allied politicians [42].

In bivariate analyses, the variations in feeling valued at the societal level were strongly associated with considerations to quit their eldercare jobs. Multivariate analyses, adjusting for an extensive set of control variables, indicated clearly that lack of recognition by the wider society had a unique and substantial impact on eldercare workers’ quitting considerations. Thus, in correspondence with the hypothesis, recognition at the societal level appears to influence turnover intentions over and above the effects of distressing work conditions, financial strain due to low pay, workplace recognition, and other known determinants.

In spite of widespread feelings of being disregarded by the wider society, many eldercare workers will stay on in their jobs. A partial explanation is probably that an overwhelming majority felt “very much/quite much valued” by recipients and work colleagues (Table 1). Supportive experiences in one’s immediate work environment may be especially important for job satisfaction and overshadow perceived lack of recognition from distal societal agents. It can be added that selective turnover is a likely reason that so few reported “not at all valued” at their workplace. Those feeling poorly valued by recipients and colleagues would quit quite soon and therefore be few in a cross-sectional sample of employed eldercare workers.

### Implications

Previous analyses of retention difficulties in eldercare have often pointed to lack of recognition of eldercare work by the wider society (e.g., [1, 35]), but studies which attempt to *quantify* this effect are scarce. The specific contribution of the present study is to demonstrate that lack of recognition at the societal level, exemplified by top municipal leaders, mass media, and the general public, plays an independent and substantial role for the retention challenge in Nordic eldercare. The findings implicate that *both* the concrete aspects of eldercare work such as working conditions, pay levels, and social relations at the workplace, *and* the perceptions of being recognised and valued at the societal level, influence staff retention. Thus, although recognition at the societal level overlaps with the more tangible aspects of eldercare work, recognition is not reducible to such tangible aspects. Rather, recognition has an autonomous role which is related to feelings of worth, self-respect, and overall esteem in society, aside from the material aspects of eldercare work [21]. A policy implication is that although improving working conditions, wages, and work-life balance is definitely important for reducing retention problems, such improvements in themselves will hardly be sufficient for sustaining a stable eldercare workforce. It is also necessary to modify the *recognition order* in society [20] which underlies the misrecognition of eldercare work.

How could society’s recognition order be changed? For decades, the valorisation of care work has been an issue in care policies as well as in women’s struggles. As mentioned above, various countries have initiated image-building public campaigns aiming at improving the prestige and attractiveness of care work [1]. In the Nordic welfare states, two state-centred approaches have been prominent [18, 43]. One is to professionalise care work and establish that care work is *qualified* labour which requires knowledge, skills, and often formal education. The other is institutionalisation, which – at least in the Nordic countries – typically implies state responsibility and public funding. These approaches have led to a transformation of eldercare, but – as shown by this study – lack of recognition of eldercare work at the societal level has remained widespread. Actually, critics claim that recent policies in the Nordic countries may have contributed to further misrecognition of eldercare work, since reforms inspired by New Public Management ideas, involving standardisation and rigid regulations of care work, have tended to undermine the autonomy of care workers [17, 35].

Thus, professionalisation and institutionalisation have not restructured the recognition order in any fundamental way. Dahl [17, 18] has proposed additional strategies which are also embraced by the aforementioned OECD report [1]: “caring for the carer” (intensified efforts to improve working conditions, pay levels, and other tangible aspects of care work) and “degendering care” (recruitment of male care workers). Such strategies may improve societal recognition of eldercare, but progress is slow, as indicated by the overwhelming proportion of women (96 %) in the samples of eldercare workers analysed in this study.

Misrecognition of eldercare work at the societal level is an obstacle against building a sustainable eldercare workforce, and future research will have to examine the effectiveness of the various strategies. Well-directed policies will be helpful, but it is probably misleading to assume that the problem can be “solved” by politicians. Lack of recognition of eldercare work is embedded in current societies and sustained in many ways, for instance by prevailing ideas about care as “dirty work” [44] and by recurrent mass media reports about the “problems” in the eldercare sector [45]. This study was made with data collected years before the Covid-19 pandemic. A pertinent topic is whether societal recognition of eldercare work has improved, or deteriorated, during the pandemic. Raising the societal recognition of eldercare work is likely to reduce the retention challenge. The goal should be that eldercare staff have no longer reasons to demonstrate under banners with the text “Union of disregarded workers”, as happened in Denmark in the early 2000 s [17].

### Limitations

Unionisation is high in all four countries; in Sweden, for instance, around 80 % of all care workers are estimated to be trade union members [36]. This suggests that the gross samples are quite representative for frontline eldercare staff, but due to lower unionisation among temporary and part-time employed, the samples will primarily be representative for well-established and committed eldercare workers. The response rate of 55 % implies a possibility of biased attrition; since data on non-responders are not available, this could not be further explored.

Since *actual* turnover is difficult to analyse without longitudinal data, studies often use turnover intentions as a proxy for staff instability, as is the case also in the present study. Although turnover intent correlates with subsequent quitting [37, 38], the statement “seriously considered to quit” must be considered an imprecise indicator of actual turnover [46, 47]. Quitting presupposes search for work and job openings (if not leaving for retirement), and the range of relevant job alternatives may be limited for many eldercare workers.

Respondents’ answers are subjective, but the relevant issue in the present study is not how societal sources actually view eldercare work, but how eldercare workers *perceive* recognition by the wider society. As the multivariate analyses adjust for a wide range of controls, the main results are not likely to be contaminated by subjective inclinations to express negative evaluations. Some findings invite further studies, for instance the country differences in feeling valued by mass media and by the general public.

## Conclusions

Lack of social recognition by the wider society is widely felt by Nordic eldercare workers. Among the substantial part of these workers who perceived being “not at all valued” at the societal level, represented by top municipal leaders, mass media, and the general public, a high proportion reported serious considerations to quit their job. Effects of lack of recognition from societal sources remained in multivariate analyses which adjusted for a series of well-known turnover determinants. Perceptions of social recognition at the societal level are an independent and significant source of instability in eldercare staff. Staff retention in eldercare can be improved by raising societal recognition of care workers, and care policies can be helpful in this respect, but the negative reputation of eldercare work is probably institutionalised in many ways and will therefore be slow to change.

## Supplementary Information

**Additional file 1: Appendix Table 1. **The 2015 Nordcare survey samples. **Appendix Table 2. **Linear probability model; “considerations to quit” (no = 0, yes = 1) regressed on perceptions of recognition by mass media and control variables, unstandardized regression coefficients (B) and 95% confidence intervals.

## Data Availability

The Nordcare 2015 dataset used by this study was collected by a Nordic research project led by professor Marta Szebehely <Marta.Szebehely@socarb.su.se>, Stockholm University, and funded by the Swedish Council for Working Life and Social Research. The project was approved by the Regional Ethical Review Board in Stockholm in 2015 (Dnr 2015/1125-31/5). The data are not publicly available, but can be obtained from the project leader or from the corresponding author on reasonable request.

## References

[1] OECD (2020). Who cares? Attracting and retaining care workers for the elderly. OECED Health Policy Studies.

[2] Scheffelaar A, Bos N, Hendriks M, van Dulmen S, Luijkx K. Determinants of the quality of care relationships in long-term care - a systematic review. Bmc Health Serv Res. 2018;18:903. 10.1186/s12913-018-3704-7 PMC6264609 30486821

[3] Castle NG, Engberg J, Men A (2007). Nursing home staff turnover: Impact on nursing home compare quality measures. Gerontologist.

[4] Trydegard GB (2012). Care work in changing welfare states: Nordic care workers’ experiences. Eur J Ageing.

[5] Castle NG, Degenholtz H, Rosen J. Determinants of staff job satisfaction of caregivers in two nursing homes in Pennsylvania. Bmc Health Serv Res. 2006;6:60. 10.1186/1472-6963-6-60 PMC1524956 16723022

[6] Faber A, Giver H, Stroyer J, Hannerz H (2010). Are low back pain and low physical capacity risk indicators for dropout among recently qualified eldercare workers? A follow-up study. Scand J Public Health.

[7] Clausen T, Tufte P, Borg V (2014). Why are they leaving? Causes of actual turnover in the Danish eldercare services. J Nurs Manage.

[8] Gerritsen DL, van Beek APA, Woods RT (2019). Relationship of care staff attitudes with social well-being and challenging behavior of nursing home residents with dementia: a cross sectional study. Aging Ment Health.

[9] Ede L, Rantakeisu U (2015). Managing Organized Insecurity: The Consequences for Care Workers of Deregulated Working Conditions in Elderly Care. Nord J Working Life.

[10] Cheng ZM, Nielsen I, Cutler H (2019). Perceived job quality, work-life interference and intention to stay Evidence from the aged care workforce in Australia. Int J Manpower.

[11] Zeytinoglu IU, Denton M, Davies S, Plenderleith JM (2009). Casualized employment and turnover intention: Home care workers in Ontario, Canada. Health Policy.

[12] Theobald H, Szebehely M, Saito Y, Ishiguro N (2018). Marketisation policies in different contexts: Consequences for home-care workers in Germany, Japan and Sweden. Int J Soc Welf.

[13] Lundmark R, Nordin M, Yepes-Baldo M, Romeo M, Westerberg K (2021). Cold wind of change: Associations between organizational change, turnover intention, overcommitment and quality of care in Spanish and Swedish eldercare organizations. Nurs Open.

[14] Hebson G, Rubery J, Grimshaw D (2015). Rethinking job satisfaction in care work: looking beyond the care debates. Work Employ Soc.

[15] Tufte P, Clausen T, Nabe-Nielsen K (2012). Client-related work tasks and meaning of work: results from a longitudinal study among eldercare workers in Denmark. Int Arch Occ Env Hea.

[16] Gaudenz C, De Geest S, Schwendimann R, Zuniga F (2019). Factors Associated With Care Workers’ Intention to Leave Employment in Nursing Homes: A Secondary Data Analysis of the Swiss Nursing Homes Human Resources Project. J Appl Gerontol.

[17] Dahl HM (2009). New Public Management, care and struggles about recognition. Crit Soc Policy.

[18] Dahl HM (2010). An old map of state feminism and an insufficient recognition of care. Nordic J Femin Gender Res.

[19] Honneth A, Fraser N, Honneth A (2003). Redistribution as recognition: A response to Nancy Fraser. Redistribution or recognition? A political-philosophical exchange.

[20] Fraser N, Fraser N, Honneth A (2003). Social justice in the age of identity politics: Redistribution, recognition, and participation. Redistribution or recognition? A political-philosophical exchange.

[21] Austen S, Jefferson T, Ong R, Sharp R, Lewin G, Adams V (2016). Recognition: applications in aged care work. Camb J Econ.

[22] Bjarnason T. Social Recognition and Employees’ Organizational Support. The Impact of Social Recognition on Organizational Commitment, Intent to Stay, Service Effort, and Service Improvements in an Icelandic Service Setting. PhD Dissertation. Göteborg: Gothenburg University, Department of sociology; 2009.

[23] Tweedie D, Wild D, Rhodes C, Martinov-Bennie N (2019). How Does performance management affect workers? Beyond human resource management and its critique. Int J Manage Rev.

[24] FHRC. 16 Effective Ways To Incorporate Employee Recognition For Enhanced Motivation: Forbes Human Resources Council, 2020. https://www.forbes.com/sites/forbeshumanresourcescouncil/2020/05/08/16-effective-ways-to-incorporate-employee-recognition-for-enhanced-motivation/?sh=660fed74126b. Accessed 8 Jan 2021.

[25] Preciate. 103 examples of workplace recognition to boost employee engagement. https://blog.preciate.com/103-examples-workplace-recognition. Accessed 28 Jan 2021.

[26] Tang JHC, Hudson P (2019). Evidence-Based Practice Guideline Nurse Retention for Nurse Managers. J Gerontol Nurs.

[27] Tran D, Hall LM, Davis A, Landry MD, Burnett D, Berg K, et al. Identification of recruitment and retention strategies for rehabilitation professionals in Ontario, Canada: results from expert panels. Bmc Health Serv Res. 2008;8:249. 10.1186/1472-6963-8-249 PMC2636785 19068134

[28] Hegney D, McCarthy A, Rogers-Clark C, Gorman D (2002). Retaining rural and remote area nurses - The Queensland, Australia experience. J Nurs Admin.

[29] Keane S, Lincoln M, Smith T. Retention of allied health professionals in rural New South Wales: a thematic analysis of focus group discussions. Bmc Health Serv Res. 2012;12:175. 10.1186/1472-6963-12-175 PMC3479013 22726758

[30] Scanlan JN, Still M. Relationships between burnout, turnover intention, job satisfaction, job demands and job resources for mental health personnel in an Australian mental health service. Bmc Health Serv Res. 2019;19:62. 10.1186/s12913-018-3841-z PMC6343271 30674314

[31] Willis-Shattuck M, Bidwell P, Thomas S, Wyness L, Blaauw D, Ditlopo P. Motivation and retention of health workers in developing countries: a systematic review. Bmc Health Serv Res. 2008;8:247. 10.1186/1472-6963-8-247 PMC2612662 19055827

[32] Svensson LG, Eriksson YU, Yrkesstatus (2009). En sociologisk studie av hur yrken uppfattas och värderas [Occupational Status. A Sociological Study on the perceptions and valuations of occupations] Research Report No 140.

[33] Banerjee A, Daly T, Armstrong P, Szebehely M, Armstrong H, Lafrance S (2012). Structural violence in long-term, residential care for older people: Comparing Canada and Scandinavia. Soc Sci Med.

[34] Moriarty J, Manthorpe J, Harris J (2018). Recruitment and retention in adult social care services.

[35] Szebehely M, Meagher G (2018). Nordic eldercare - Weak universalism becoming weaker?. J Eur Soc Policy.

[36] Stranz A, Szebeley M. Organizational trends impacting on everyday realities. In: Kristensen K, Pilling D, editors. The Routledge Handbook of Social Care Work Around the World. London: Routledge, 2018. https://www.routledgehandbooks.com/doi/10.4324/9781315612805-4. Accessed 6 Jan 2021.

[37] Sverke M, Hellgren J, Näswall K (2002). No Security: A Meta-Analysis and Review of Job Insecurity and Its Consequences. Journal of Occupational Health Psychology.

[38] Cohen G, Blake RS, Goodman D (2016). Does Turnover Intention Matter? Evaluating the Usefulness of Turnover Intention Rate as a Predictor of Actual Turnover Rate. Rev Public Pers Adm.

[39] Hellevik O (2009). Linear versus logistic regression when the dependent variable is a dichotomy. Qual Quant.

[40] Mood C (2010). Logistic Regression: Why We Cannot Do What We Think We Can Do, and What We Can Do About It. Eur Sociol Rev.

[41] Von Hippel P. Linear vs. Logistic Probability Models: Which is Better, and When? 2015. Available at https://statisticalhorizons.com/linear-vs-logistic. Accessed 3 Feb 2021.

[42] Andersson LM (1996). Employee cynicism: An examination using a contract violation framework. Hum Relat.

[43] Hernes H (1987). Welfare state and woman power. Essays in state feminism.

[44] Twigg J (2000). Carework as a form of bodywork. Ageing Soc.

[45] Carlstedt E, Jonson H. Amazing numbers and bottom rankings: the reporting of nursing home resident user surveys in the press. Int J Sociol Soc Pol. 2021. Online publication 2 Feb 2021. 10.1108/IJSSP-07-2020-0266.

[46] Holtom BC, Mitchell TR, Lee TW, Eberly MB (2008). Turnover and Retention Research: A Glance at the Past, a Closer Review of the Present, and a Venture into the Future. Acad Manag Ann.

[47] Sjoberg A, Sverke M (2000). The interactive effect of job involvement and organizational commitment on job turnover revisited: a note on the mediating role of turnover intention. Scand J Psychol.

