# Supplementary material for: Lack of recognition at the societal level heightens turnover considerations among Nordic eldercare workers: a quantitative analysis of survey data
Source: BMC Health Serv Res. 2021 Jul 27;21:747. doi: 10.1186/s12913-021-06734-4 (PMC8315089; doi:10.1186/s12913-021-06734-4)
Supplement: Supplementary file 1 — Additional file 1: Appendix Table 1. The 2015 Nordcare survey samples. Appendix Table 2. Linear probability model; “considerations to quit” (no = 0, yes = 1) regressed on perceptions of recognition by mass media and control variables, unstandardized regression coefficients (B) and 95% confidence intervals. [file 12913_2021_6734_MOESM1_ESM.docx]

Appendix Table 1 The 2015 Nordcare survey samples

Sample	Denmark	Finland	Norway	Sweden
Total number of respondents	1016	972	920	769
Seriously considered to quit (%)	41.9	38.2	33.5	52.3
Women (%)	96.4	96.2	96.2	92.6
Age category				
- age up to 39 years %	17.7	29.8	16.9	26.2
- age 40-54 years %	43.3	39.4	38.9	45.2
- age 55+ years %	39.0	30.8	44.1	28.6
Immigrant %	8.5	2.8	10.5	31.7
Years in school//general education				
- up to 12 years %	69.9	31.0	44.8	55.5
- 13-14 years %	19.7	38.9	41.0	29.3
- 15+ years %	10.4	30.1	14.2	15.2
Schools/education in care work				
- less than 1 year %	12.5	6.0	22.9	20.7
- 1 years %	46.1	22.6	32.5	39.7
- 2+ years %	41.4	71.4	44.6	39.7
Experience in eldercare work				
- 0-5 years %	12.9	26.5	8.9	18.3
- 6-9 years %	15.3	16.7	11.1	13.8
- 10-19 years %	33.1	28.6	32.0	33.4
- 20+ years %	38.8	28.2	48.0	34.5
Experiencing financial strain				
- no, little %	73.8	38.4	62.1	42.1
- quite much %	16.8	31.5	24.4	31.6
- yes, very much %	9.4	30.1	13.5	26.3
Work-life balance?				
- very good %	54.3	17.5	28.6	16.4
- fairly good %	37.4	51.5	55.6	45.2
- not good, bad %	8.3	30.9	15.8	38.4
Unsatisfactory work conditions experienced daily or weekly, average 8 items, range 0–8 (st.dev.)	2.95 (2.23)	4.13 (1.94)	3.17 (2.18)	3.32 (2.17)
Experiencing harassment from recipients or relatives daily or weekly, average 4 items (st.dev.)	0.41 (0.77)	0.70 (0.90)	0.38 (0.73)	0.41 (0.78)
Self-rated health very good or good %	68.8	62.7	73.2	58.1
Self-rated health below good %	31.2	37.3	26.8	41.9
Variation in number of valid answers	990 -1016	956 - 972	890 - 920	744 - 762

Appendix Table 2 Linear probability model; “considerations to quit” (no = 0, yes = 1) regressed on perceptions of recognition by mass media and control variables, unstandardized regression coefficients (B) and 95% confidence intervals

	B	95%CI
Perceived recognition from mass media		
Very/quite much valued (ref.)		
Not much valued	0.019	-0.029/0.068
Not at all valued	**0.083**	0.032/0.134
Don’t know	0.037	-0.013/0.087
Not much valued by recipients	0.018	-0.035/0.072
Not much valued by work colleagues	**0.114**	0.069/0.158
Danish sample (ref.)		
Finland	**-0.213**	-0.259/-0.166
Norway	**-0.114**	-0.157/-0.070
Sweden	-0.014	-0.062/0.034
Men (ref: women)	0.058	-0.013/0.130
Age up to 39 years	**0.118**	0.075/ 0.160
Age 40-54 years (ref.)		
Age 55+ years	**-0.059**	-0.094/-0.023
Immigrant (ref. not immigrant)	-0.089	-0.138/-0.040
General education up to 12 years (ref.)		
13-14 years education	0.031	-0.004 /0.066
15+ years education	0.013	-0.030/0.057
Education in care work < 1 year	-0.050	-0.097/0.004
Education in care work 1-2 years	-0.041	-0.075/-0.007
Education in care work 2+ years (ref.)		
Eldercare work for 0-5years	-0.030	-0.078/-0.018
Eldercare work 6-9 years	0.012	-0.036/0.059
Eldercare work 10-19 years (ref.)		
Eldercare work 20+ years	0.028	-0.010/0.066
No/little financial strain (ref.)		
Quite much financial stress	**0.066**	0.029/0.103
Very much financial stress	**0.113**	0.070/0.156
Very good work-life balance	**-0.063**	-0.100/-0.027
Fairly good (ref.)		
Not good, bad	**0.142**	0.103/0.182
Index work conditions (centered around mean)	**0.038**	0.031/0.046
Index harassment (centered around mean)	0.025	0.005/0.045
Self-rated health very good/good (ref.)		
Self-rated health below good	**0.119**	0.087/0.151
Constant	0.355	
Adjusted Rsquare	0.186	
Number of respondents		3,527

Coefficients in **bold** = p-value < 0.01.
